# Double strand break repair by capture of retrotransposon sequences and reverse-transcribed spliced mRNA sequences in mouse zygotes

**DOI:** 10.1038/srep12281

**Published:** 2015-07-28

**Authors:** Ryuichi Ono, Masayuki Ishii, Yoshitaka Fujihara, Moe Kitazawa, Takako Usami, Tomoko Kaneko-Ishino, Jun Kanno, Masahito Ikawa, Fumitoshi Ishino

**Affiliations:** 1 Division of Cellular and Molecular Toxicology, Biological Safety Research Centre, National Institute of Health Sciences (NIHS), 1-18-1 Kamiyoga, Setagaya-ku, Tokyo, 158-8501, Japan; 2 Department of Epigenetics, Medical Research Institute, Tokyo Medical and Dental University, 1-5-45 Yushima, Bunkyo-ku, Tokyo 113-8510, Japan; 3 Research Institute for Microbial Diseases, Osaka University, Suita, Osaka 565-0871, Japan; 4 Facility for Recombinant Mice, Medical Research Institute, Tokyo Medical and Dental University, 2-3-10 Kandasurugadai, Chiyoda-ku, Tokyo 101-0062, Japan; 5 School of Health Sciences, Tokai University, 143 Shimokasuya, Isehara, Kanagawa 259-1193, Japan; 6 Global Centre of Excellence Programme for International Research Centre for Molecular Science in Tooth and Bone Diseases, Tokyo Medical and Dental University, 1-5-45 Yushima, Bunkyo-ku, Tokyo 113-8510, Japan

## Abstract

The CRISPR/Cas system efficiently introduces double strand breaks (DSBs) at a genomic locus specified by a single guide RNA (sgRNA). The DSBs are subsequently repaired through non-homologous end joining (NHEJ) or homologous recombination (HR). Here, we demonstrate that DSBs introduced into mouse zygotes by the CRISPR/Cas system are repaired by the capture of DNA sequences deriving from retrotransposons, genomic DNA, mRNA and sgRNA. Among 93 mice analysed, 57 carried mutant alleles and 22 of them had long *de novo* insertion(s) at DSB-introduced sites; two were spliced mRNAs of *Pcnt* and *Inadl* without introns, indicating the involvement of reverse transcription (RT). Fifteen alleles included retrotransposons, mRNAs, and other sequences without evidence of RT. Two others were sgRNAs with one containing T7 promoter-derived sequence suggestive of a PCR product as its origin. In conclusion, RT-product-mediated DSB repair (RMDR) and non-RMDR repair were identified in the mouse zygote. We also confirmed that both RMDR and non-RMDR take place in CRISPR/Cas transfected NIH-3T3 cells. Finally, as two *de novo* MuERV-L insertions in C57BL/6 mice were shown to have characteristic features of RMDR in natural conditions, we hypothesize that RMDR contributes to the emergence of novel DNA sequences in the course of evolution.

Whole genome sequencing of a number of different mammalian species has established that approximately 50% of the mammalian genome is derived from transposable elements^1^,^2^,^3^. Retrotransposons, which mobilize via an RNA intermediate by a copy-and-paste mechanism, comprise the majority of mammalian transposable elements, whereas DNA transposons, which move via a cut-and-paste mechanism, comprise a relatively small fraction, having accumulated mutations that render them immobile^4^. Retrotransposons can be subdivided into two classes, long terminal repeat (LTR) retrotransposons and non-LTR retrotransposons^4^,^5^. LTR retrotransposons contain two LTRs, they encode the proteins Gag and Pol with activities similar to those of simple retroviruses such as protease, reverse transcriptase and integrase activities, but they lack an envelope (*Env*) gene. The LTRs are direct sequence repeats that contain a promoter recognized by the host RNA polymerase II to produce the retrotransposon mRNA. The Gags are structural proteins that form the virus-like particle, inside of which RT takes place. The reverse transcriptase copies its mRNA into a cDNA, and the integrase inserts the cDNA into a new target site. The cleavages of the two strands at the target site are staggered, resulting in target-site duplications (TSDs)^4^,^5^. Long interspersed elements (LINEs), a type of non-LTR retrotransposon, lack LTRs and encode two open reading frames (ORFs). These elements mobilize by target-site-primed reverse transcription (TPRT). TPRT is a mechanism by which an element-encoded endonuclease generates a single-strand nick in the genomic DNA, liberating a 3′OH that is used to prime reverse transcription of the RNA. The integration site formed by TPRT is usually flanked by TSDs^4^,^5^. The endonuclease and reverse transcriptase activities of non-LTR retrotransposons also have the ability to mobilize other non-autonomous short interspersed elements (SINEs)^6^,^7^,^8^, and certain classes of non-coding RNAs^9^,^10^,^11^,^12^ and mRNAs^13^,^14^, which can result in the formation of processed pseudogenes.

Retrotransposons continue to sculpt mammalian genomes and behave as insertional mutagens, either by disrupting exons or by insertion into introns, leading to mis-splicing^5^,^15^,^16^. Hence, retrotransposons occasionally have deleterious effects on host genes and thus organisms. However, a growing body of evidence suggests that retrotransposons and retrotransposon-derived genes have also acquired functions essential for host survival during mammalian evolution including placental formation, neurogenesis and gene regulation^17^,^18^,^19^,^20^,^21^,^22^,^23^,^24^,^25^.

Recent studies have shown that a large number of different retrotransposon families are highly transcribed in the mouse zygote, and in fact, they produce cDNAs by reverse transcription (RT)^26^,^27^,^28^,^29^. In *Saccharomyces cerevisiae,* the capture of Ty1 retrotransposon cDNA at the site of DSBs has been observed when homologous recombination is blocked^30^,^31^. It was thus reasoned that RT-product-mediated DSB repair (RMDR) is functional in the mouse zygote because of its high RT activity.

DSBs result from both exogenous insults (e.g., reactive oxygen species, irradiation, chemical agents and ultraviolet light) and endogenous cellular events (e.g., transposition, meiotic double strand break formation)^5^,^32^,^33^. The clustered regularly interspaced short palindromic repeat (CRISPR)/Cas system has made it possible to induce double strand breaks (DSBs) at specific loci in the mammalian genome^34^,^35^,^36^,^37^,^38^,^39^. In this report, DSBs were introduced into 8 genomic loci of the mouse zygote, the *Peg10-ORF1* and *Peg10-ORF2* regions and the *Cxx1a*, *Cxx1b*, *Rgag1, Rsph6a*, *Spaca5* and *Ddx3y* genes. We analysed the sequences of the DSB-induced sites to determine whether RMDR was at work. We also introduced DSBs into NIH-3T3 cells to assess the universality of the phenomenon, doing so with or without an RT-inhibitor to determine whether RMDR could be inhibited.

## Results

### DSBs were introduced by CRISPR/Cas into mouse zygotes

**Figure 1:**
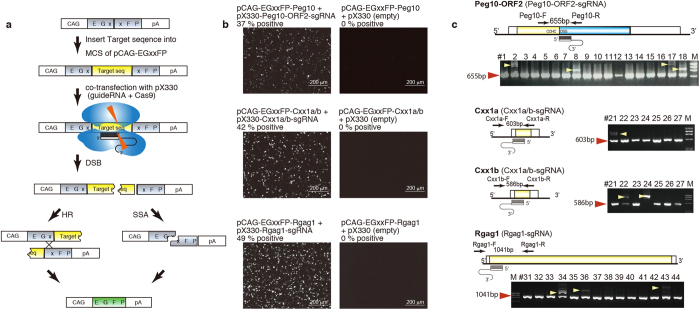
CRISPR/Cas mediated gene manipulation. (**a**) The pCAG-EGxxFP plasmid contains 5′ and 3′ EGFP fragments that share 482 bp under a ubiquitous CAG promoter. A 500-bp genomic fragment containing the sgRNA target sequence was placed between the EGFP fragments of the pCAG-EGxxFP plasmid. The resulting target plasmid was co-transfected with pX330 plasmids expressing the sgRNA and hCas9 into HEK293T cells. Once the target sequence was digested by sgRNA guided CAS9 endonuclease, homology dependent repair (HR: homologous recombination, or SSA: single-strand annealing) took place and reconstituted the EGFP expression cassette. MCS; multi cloning site. (**b**) The DSB efficiency was validated with the pCAG-EGxxFP system by observing EGFP fluorescence 48 hrs after the transfection (scale bar: 200 μm). The percentages of EGFP-positive cells are indicated. (**c**) Schematic representation of the positions of each sgRNA and primer to check the CRISPR/Cas mediated mutations (left side of (**c**)). Electrophoresis of the PCR products from each of the pX330 plasmid-injected mice (the right side of (**c**)). At least four PCR products (yellow arrowheads) were larger than expected (WT: red arrowheads) in the *Peg10*-ORF2-sgRNA-injected mice. One and two PCR products (yellow arrowheads) were larger than *Cxx1a* WT and *Cxx1b* WT, respectively, in the *Cxx1a/b*-sgRNA-injected pups. Three PCR products (yellow arrowheads) were larger than 1041 bp (*Rgag1* WT) in the *Rgag1*-sgRNA-injected pups.

Single guide RNAs (sgRNAs) were designed for each of the eight target genomic loci, and the DSB induction efficiency was validated with an EGxxFP system^40^ (Fig. 1a). We confirmed that all the sgRNAs were able to induce fluorescent cells at more than 30% efficiency (Fig. 1b, Supplementary Fig. 1). It was previously reported that more than 30% efficiency obtained with an EGxxFP system allows for the stable generation of mutant mice by injecting the sgRNA sequence for each gene along with the hCas9 gene as an RNA or a plasmid (with oligo DNAs for the knock-in mice) into fertilized eggs^40^. Mutant and knock-in mice were obtained by CRISPR/Cas injection under various conditions (Supplementary Table 1,2). The pups and embryos that developed from these embryos were subjected to PCR and subsequent sequence analysis (Fig. 1c, Supplementary Table 1,2). CRISPR/Cas-mediated mutant mice (including mosaicism mutations) were obtained with high efficiency (23.1% to 100%) from all the different sgRNAs used (Fig. 1b, Supplementary Fig. 1). In total, 61% (57 out of 93) of the embryos or pups carried a CRISPR/Cas-mediated mutant allele, suggesting that the DSB induction activity is adequate in these mouse zygotes and validating the EGxxFP system (Supplementary Table 1,2, Fig. 1b, Supplementary Fig. 1). However, we found that 22 pups and embryos had extra unknown PCR products larger than the expected length (Fig. 1c, Supplementary Table 1,2). We isolated these extra PCR products after electrophoresis, and 20 out of 22 PCR products had their sequences successfully determined (Supplementary Table 1,2). These PCR products were found to have *de novo* insertions of retrotransposons, genomic DNA, mRNA and sgRNA sequence at the DSB-induced loci. These data demonstrate that, at least in the case of two insertions of mRNA sequences, i.e., *Pcnt* and *Inadl*, which are missing introns, RMDR is functional in mouse zygotes.

### DSBs were repaired by the capture of retrotransposon sequences

**Figure 2:**
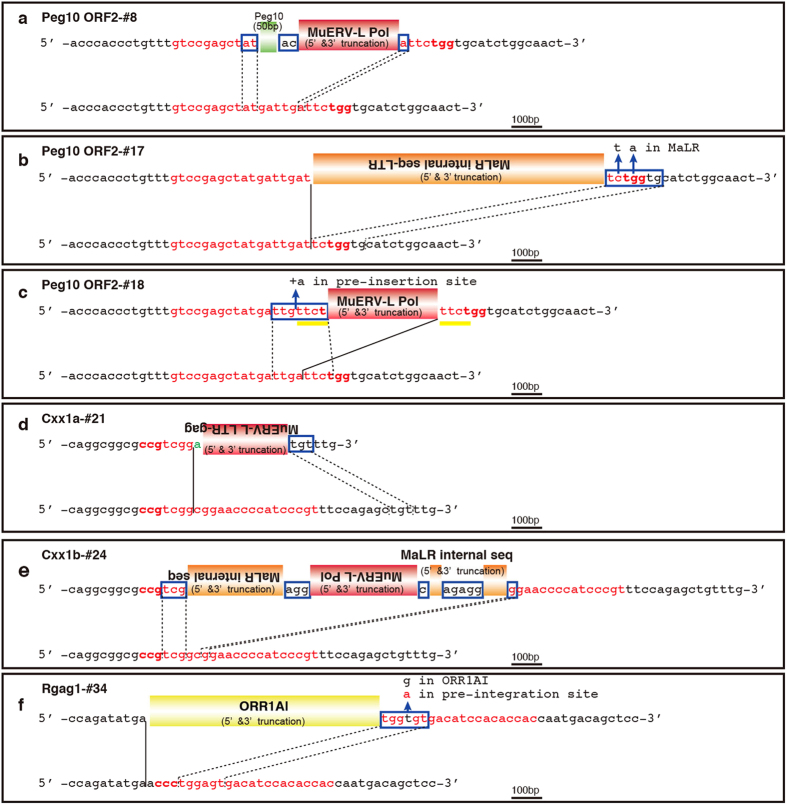
Structure of the captured retrotransposons associated with DSB repair. *De novo* inserted retrotransposons at the *Peg10*-ORF2 (**a**–**c**), *Cxx1a/b* (**d**,**e**), and *Rgag1* (**f**) loci were induced by pX330 injection into mouse zygotes. Both the post-integration site and pre-integration sequences (bottom of the panel) are shown. The nucleotide sequences that correspond to the single guide RNA sequence and the PAM sequences are shown in red and bold red characters, respectively. The black lines indicate the junction sites between pre- and post-integration sequences. The sequences in the blue boxes are overlapping microhomologies and are marked with black dotted lines. Short sequences of unknown origin are shown in green. Each insertion was truncated at both the 5′ and 3′ ends, but they demonstrated distinct features. These included the absence of LTRs and TSDs. (**a**) Together with MuERV-L, 50 bp of *Peg10* cDNA sequence was inserted with 1-bp microhomology. (**b**) A truncated MaLR internal sequence was inserted with a 7-bp overlapping microhomology (a 2-bp mismatch). (**c**) A truncated MuERV-L Pol region was inserted with a 7-bp microhomology (1-bp mismatch) and 4-bp TSDs (yellow bars) ‘ttct’. (**d**) A truncated MuERV-L was inserted with 3-bp overlapping microhomology. (**e**) Multiple truncated retrotransposons were inserted with 1-5-bp microhomologies. (**f**) A truncated ORR1AI retrotransposon was inserted with a 6-bp microhomology (1-bp mismatch).

Detailed characterization of the *de novo* insertions at the target DSB sites in the *Peg10*-ORF2 coding region revealed that two of the animals (*Peg10*-ORF2-#8 and *Peg10*-ORF2-#18) had 327-bp and 357-bp insertions of the murine endogenous retrovirus-L (MuERV-L, also known as the MERVL or Erv4) Pol protein coding region^26^ (Fig. 2a,c). MuERV-L is an endogenous retrovirus that is one of the most abundant transcripts in the 2-cell stage embryo^26^,^27^,^28^,^29^. In each case, there were small overlapping nucleotides called “microhomologies” between the inserted retrotransposon and the DSB-induced target site, and truncations of both the 5′ and 3′ regions of the inserted retrotransposon including the LTRs were present, suggest that these LTR retrotransposons had not been integrated by typical replicative retrotransposition^41^,^42^,^43^,^44^,^45^,^46^. Furthermore, the *Peg10*-ORF2-#17 animal was found to have a 950-bp insertion of a partial internal region and a truncated LTR of the retrovirus-like element MaLR, which is the second most abundant retrotransposon transcript in the 2-cell stage mouse embryo^29^ (Fig. 2b).

To the best of our knowledge, this is the first direct evidence of the introduction of MuERV-L and MaLR retrotransposons at a specifically desired genomic locus in mouse zygotes. Because MaLR does not encode any known protein and its means of propagation in the genome is unknown, it has been suggested that the RT activity of MuERV-L might be the means of its propagation^27^,^28^. Insertions of partial retrotransposon sequences with microhomologies were also observed at all of the target DSB sites introduced (Fig. 2d–f, Supplementary Fig. 2a–c,e,g–j). Although the DSB-induced loci were different, the same types of endogenous retroviruses were inserted at each locus, indicating that the capture of retrotransposon sequences may occur at any DSB site in the mouse zygote. Furthermore, there was a case in which an allele had multiple retrotransposon insertions at the same DSB site (Fig. 2e). Each of the junction sequences between the retrotransposons has 1–5 bp of complete overlapping microhomology.

### DSBs were repaired by the capture of RT-mediated cDNA

**Figure 3:**
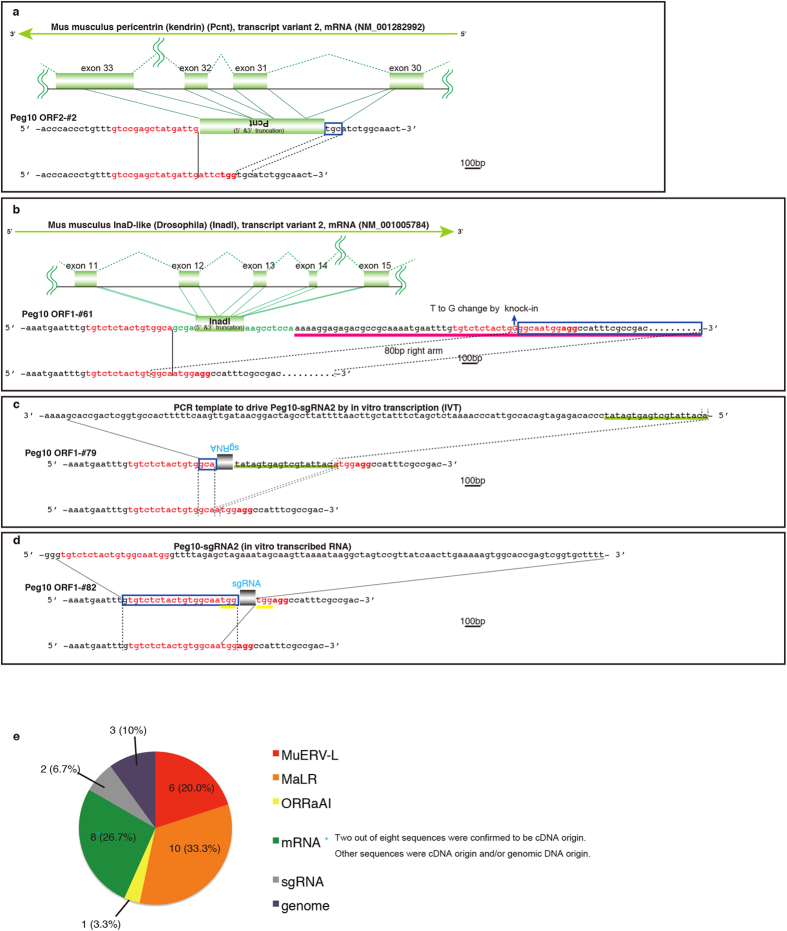
RT-product-mediated DSB repair (RMDR) and non-RT-product-mediated DSB repair (non-RMDR). (**a**) A partial sequence of the processed *Pcnt* gene (6803–7599 bp: NM_001282992) in reverse orientation was inserted into the DSB site mediated by RMDR. The integrated *Pcnt* fragment skipping introns 30–32 was inserted with a 3-bp microhomology. (**b**) A partial sequence of the processed *Inadl* gene (1527–1836 bp: NM_001005784) and a DNA oligo (pink bar) including a T to G mutation with 40 bp out of 53 bp in the 5′ homology arm were inserted into the DSB site. The 3′ side of the DSB site was repaired by HR with the long homology of the DNA oligo and 5′ side of the DSB site repaired by capture of the *Inadl* gene fragment, skipping introns 11–14 (RMDR). (**c**) A partial sgRNA sequence with T7 promoter (green bar) was inserted with 3-bp and 1-bp microhomologies mediated by non-RMDR. (**d**) A partial sgRNA in reverse orientation was inserted with a 21-bp microhomology, including a 20-bp guide RNA sequence. (**e**) Distribution of 30 insertion sequences at CRISPR/Cas DSB sites in 20 mice in which the DSBs were repaired by the capture of long DNA sequences. Approximately 57% of the inserted sequences were derived from LTR retrotransposons. The 8 insertions correspond to the exon regions of 8 genes. Two insertion sequences correspond to multiple exons, skipping introns, demonstrating that they are derived from cDNA.

**Figure 4:**
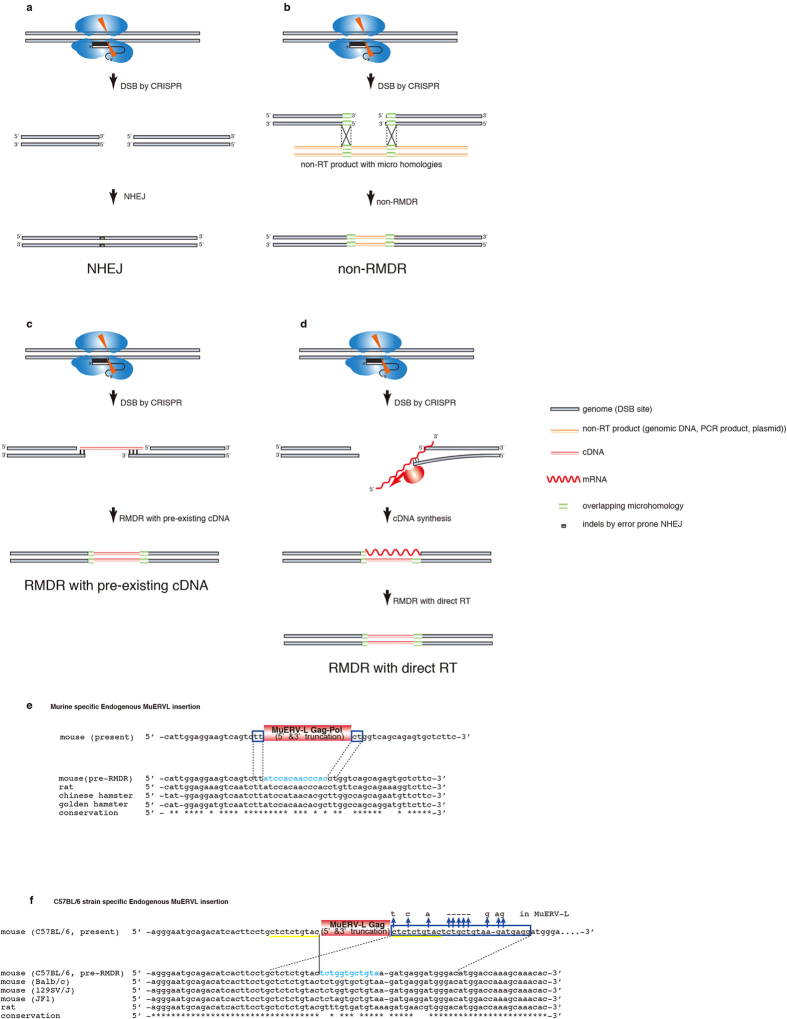
Possible mechanisms of RMDR, and RMDR under natural conditions. DSBs (the orange triangles) induced by CRISPR/CAS (blue sphere) are shown undergoing repair by NHEJ (**a**), non-RT-product-mediated DSB repair (non-RMDR) (**b**), RMDR with pre-existing cDNA (**c**) and RMDR with direct RT (**d**). Most of the DSBs induced by CRISPR/Cas are repaired by NHEJ (**a**), while certain DSBs are repaired by the capture of other sequences (**b**–**d**). (**c**) A pre-existing cDNA (red bar) generated by RT anneals with both DNA ends of a DSB site, which is repaired (RMDR with double microhomologies). (**d**) mRNA anneals with one DSB end with microhomology, and cDNA is synthesized by RT machinery. Two murine-specific truncated MuERV-L insertions were identified by comparing rodent genomes. Pre-integration sequences (indicated in blue text) were deduced from other available rodent genomes. (**e**) Murine-specific truncated MuERV-L (52824976–52825534 bp: chromosome 9) was integrated with two 2-bp microhomologies at both the 5′ and 3′ ends. (**f**) C57BL/6 strain-specific truncated MuERV-L (191922408–191922914 bp: chromosome 1) was integrated with a 27-bp microhomology (6-bp mismatches and a 5-bp insertion).

In addition to retrotransposon sequences there were also mRNA sequence insertions at the DSB-induced loci. *De novo* insertions at *Peg10* ORF2, *Peg10* ORF1 and *Spaca5* included mRNA sequence-derived partial sequences of the *Pcnt* (*Pericentrin*) gene (Fig. 3a), *Inadl* (*InaD-like*) gene (Fig. 3b), *Cpd* (*carboxypeptidase D*) gene (Supplementary Fig. 2b), *Tpm3* (*tropomyosin 3, gamma*) gene (Supplementary Fig. 2c), *Zfp609* (*zinc finger protein 609*) gene (Supplementary Fig. 2d), *Actr2* (*ARP2 actin-related protein 2*) gene (Supplementary Fig. 2j) and *Peg10* gene (Fig. 2a, Supplementary Fig. 2a). Expression of the *Pcnt, Inadl, Cpd, Tpm3, Zfp609, Actr2* and *Peg10* genes was confirmed at the 2- to 16-cell embryonic stages^29^,^47^,^48^, and the inserted partial *Pcnt* and *Inadl* sequences correspond to the 6803–7599 bp and 1527–1836 bp regions of the full length *Pcnt* and *Inadl* mRNAs, respectively, skipping *Pcnt* intron 30–32 and *Inadl* intron 11–14, demonstrating that these partial *Pcnt* and *Inadl* insertions are mediated by RMDR. The sequences flanking the insertions have no polyA tails for any of the captured genes, but short microhomology is present for *Pcnt, Cpd, Tpm3, Zfp609, Actr2* and *Peg10*, supporting the notion that the cDNA gene formation is not mediated by conventional TPRT pathways^14^,^49^,^50^,^51^,^52^ but rather by RMDR (Fig. 4c,d).

The mouse with the *Inadl* insertion was produced in the process of obtaining knock-in (KI) mice with a point mutation in the CCHC zinc finger domain of Peg10 ORF1 by CRISPR/Cas co-injection with DNA oligos. The DNA oligos have 53 bp and 80 bp homologous regions and a T to G point mutation near the DSB site. As a result, 5 pups (#53, #54, #55, #61 and #62 in Table S1) were born with the KI allele (mosaicism) and 5 pups (#52, #55, #61, #62 and #63 in Table S1) had captured DNA sequences (the sequences of #62 and #63 were not determined). In mouse #55, the KI allele and the captured allele were independent (mosaicism); however, in mouse #61, the 5′ end of the DSB-induced site was repaired by RMDR (*Inadl* mRNA) and the 3′ end of the DSB-induced site was repaired by HR at the same allele, indicating that RMDR and HR might alternatively repair DSBs (Fig. 3b). We also tried to produce KI mice in the Peg10 ORF2 DSG protease domain, however no KI mice were obtained in this experiment.

### DSBs repaired by the capture of injected DNA templates

Two *Peg10*-ORF1-sgRNA sequences were captured at DSB sites (Fig. 3c,d) by injection of *in vitro* transcribed sgRNA into mouse zygotes. One with T7 promoter sequence at its 5′ end was derived from the PCR product for *in vitro* transcription, which was not eliminated through the purification of *in vitro* transcribed sgRNA, demonstrating that non-RT-product-mediated DSB repair (non-RMDR) is also at work (Fig. 3c). Another captured sgRNA sequence was derived from PCR product (non-RMDR) or RT-mediated cDNA (RMDR) (Fig. 3d).

The global transcription level is very low at the one–cell stage until the two-cell stage^53^,^54^. Therefore, the transcription from CRISPR-Cas DNA plasmid may be very low compared with injected *in vitro* transcribed RNA, making RNA injection into the cytoplasm more efficient than plasmid injection^55^. High DSB activity might be the reason why the injected DNA templates were captured only in mice with DSBs induced by CRISPR/Cas RNA injection.

### Possible mechanism of RMDR

The data show that RMDR is at work in mouse zygotes, at least in the case of two spliced mRNAs (Fig. 3a,b). At the same time, non-RMDR is also at work in mouse zygotes, at least in one of the CRISPR/Cas RNA injected mice (Fig. 3c). These data suggest that DNA fragments in the nucleus, whether generated by RT (RMDR) or not (non-RMDR), are captured at DSB sites. In all other cases, we cannot determine whether the captured sequences were derived from RT. However, RMDR is more likely than non-RMDR because 20% of the captured sequences are derived from exons compared to only 1% of exon sequences in the whole genome. The enrichment of exons here favours the idea that they are of cDNA origin. Furthermore, *in silico* analysis shows that MuERV-L and MaLR sequences occupy only 0.04% and 0.01% of the mouse genome, respectively; however, 20% and 33.3% of captured sequences in DSB-induced mice are MuERV-L and MaLR, respectively, suggesting that the capture of MuERV-L, MaLR and mRNA sequences is most likely mediated by RMDR. Therefore, it is highly plausible that DSBs are repaired not only by classical NHEJ and HR but also by RMDR, in mouse zygotes.

Previous studies demonstrated that MuERV-L exhibits two-cell specific expression, suggesting that DSB repair by MuERV-L insertion tends to occur at the 2-cell embryonic stage or later if the capture of MuERV-L at DSB sites is in fact mediated by the RT activity of MuERV-L^26^,^27^,^28^,^29^. In this study, the amount of the inserted MuERV-L PCR band was mostly less than 30% of the total PCR products in each mouse, suggesting that RMDR occurred in a single allele of an individual cell in 2-cell or later stage embryos, whereas the MuERV-L insertion *Cxx1b*-#24 apparently occurred at the one cell embryo stage because there is no allele other than the MuERV-L insertion allele (Figs 1c,2e). The coincidence between these two events, MuERV-L and MaLR being two of the most abundantly expressed transcripts at the 2-cell stage, while MuERV-L and MaLR are two of the most frequent insertions at DSB-induced loci in this study, also indicates that the capture of MuERV-L and MaLR is mediated by transcriptional level-dependent RMDR rather than non-RMDR (Fig. 3e). There are previous reports similarly related to RMDR. They involve a DSB repair mechanism affected by the endonuclease-independent (ENi) retrotransposition of an artificial human L1 reporter^52^,^56^,^57^. These ENi retrotranspositional features include a lack of target site duplications (TSDs) and frequent truncations at both the 5′ and 3′ ends of the artificial L1 reporter in NHEJ-deficient CHO cells, but the ENi retrotranspositions have no microhomology^52^,^56^. Because RMDR is often associated with microhomology and may be mediated by an LTR-type retrotransposon, i.e., MuERV-L, the mechanism of RMDR might be different from that of ENi retrotransposition mediated by L1, a non-LTR retrotransposon. It was previously reported that mRNA in 2-cell stage embryo undergoes RT in mice^27^,^58^. Therefore, we propose RMDR with pre-existing cDNA (Fig. 4c) and RMDR with direct RT (Fig. 4d), although the detailed mechanisms of how RNA is reverse transcribed are unknown and the possibility of non-RMDR cannot be ruled out (Fig. 4b). If a cDNA becomes annealed with both of the DSB DNA ends via microhomologies, the DSB is repaired by the filling in of the missing base pairs (RMDR with a double microhomology) (Fig. 4c, Supplementary Fig. 2g). If a cDNA is annealed with only one of two DSB DNA ends, the cDNA and the other DSB end are repaired by NHEJ (RMDR with a single microhomology) (Supplementary Fig. 3a, Figs 2b–d,f,3a,d, Supplementary Fig. 2d,e,h,i). Most of the junction sequences in the multiple retrotransposons and mRNA insertions have one to five microhomologous nucleotide sequences (Fig. 2e, Supplementary Fig. 2a,j), indicating that these multiple insertions were mediated by sequential-RMDR (s-RMDR) (Supplementary Fig. 3b,c). Although we were unable to firmly establish the mechanism without performing further experiments, there does exist a capture process of retrotransposons and/or mRNA sequences at DSB sites in mouse zygote RMDR.

### RMDR could be inhibited by an RT inhibitor in cultured cells

Because two cDNAs with skipped introns were inserted into DSB-induced sites, it is clear that at least 2 of the 30 captured sequences were mediated by RMDR. To assess the possibility that the other insertions were also from cDNA by RT, we introduced DSBs into an NIH-3T3 cell line by transfecting a CRISPR/Cas plasmid (pX330-Peg10-ORF1, including both sgRNA and hCas9 genes) and a pTracer-CMV/Bsd plasmid (including the Blasticidin S resistance gene), and performing Blasticidin S drug selection, with or without the RT inhibitor azidothymidine (AZT), which is known to inhibit human and mouse L1 retrotransposition in HeLa cells^59^.

Extra unidentified PCR products larger than the expected length were observed in CRISPR-Cas transfected cells regardless of the presence of the RT-inhibitor. The ratio of these extra products was reduced by the addition of AZT to the culture medium (Supplementary Fig. 4a–c). Sequencing analysis of the extra PCR bands revealed that insertions of mRNA, retrotransposons, and transfected plasmids (both pX330-Peg10-ORF1 and pTracer-CMV/Bsd) were observed in the absence of AZT (Supplementary Fig. 5), whereas insertions of only plasmid DNA and genomic DNA sequences were observed with AZT (Supplementary Fig. 6). The capture of plasmids (both pX330-Peg10-ORF1 and pTracer-CMV/Bsd) was observed in 35.4% (without AZT) and 75% (with AZT), and the inserted regions include the plasmid vector backbone (not the gene body), suggesting that it is mediated by non-RMDR (Supplementary Fig. 7). One of the cDNA insertion sequences without AZT was the *Tubulin folding cofactor B* (*Tbcb*) gene, skipping introns 1–3, demonstrating that RMDR occurs not only in mouse zygotes but also in cultured cells (Supplementary Fig. 5f). Approximately 53% of the captured sequences were occupied with MuERV-L and MaLR retrotransposons in mouse zygotes, while the retrotransposons in the NIH-3T3 cell line included L1 (12.9%) and ERV1/2 (12.9%) (Fig. 3e, Supplementary Fig. 7a). This difference in the species of incorporated retrotransposons might reflect their cell type-specific expression levels. The capture of plasmid DNA was not influenced by AZT, suggesting a non-RMDR mechanism. In any case, DNA fragments in the nuclei, whether generated by RT (RMDR) or not (non-RMDR), are captured at DSB sites. The reason why plasmid DNA is not captured in mouse zygotes may be that the zygote has sufficiently high RT activity to produce an excessive amount of cDNA compared to the exogenous plasmid DNA.

### RMDR could be functional under natural conditions

Finally, to determine whether RMDR occurs under natural conditions, we screened potential MuERV-L insertions in the mouse genome. As it is necessary to predict the pre-integration DNA sequence to identify microhomologies, two murine-specific MuERV-L insertions with both 5′ and 3′ truncations were identified by comparative analysis of rodent genomes. One insertion was a murine-specific truncated Gag-Pol region of MuERV-L with two 2-bp overlapping microhomologies at both DSB ends (Fig. 4e). The other insertion was a C57BL/6 mice strain-specific truncated Gag region of MuERV-L with a 27-bp microhomology (6-bp mismatches and 5-bp insertion) (Fig. 4f). Although this insertion has 10-bp TSDs, these TSDs were not generated by endogenous MuERV-L integrase activity, but by other DSB events. This is because MuERV-L retrotranspositions cause random 5 bp (rarely 6 bp) TSDs when they retrotranspose (Supplementary Table 3). As these insertional features are identical with those of RMDR, we hypothesize that RMDR contributes to the emergence of novel DNA sequences in the course of evolution.

## Discussion

It is perhaps one of the greatest mysteries of biological evolution how retrotransposons, endogenous retroviruses (ERVs) and their remnant DNA sequences have come to occupy one half of the mammalian genome. Recently, these sequences have drawn attention as one of the ostensible driving forces of genomic evolution^17^,^18^,^19^,^20^,^21^,^22^,^23^,^24^. In this report, we demonstrated that DSBs introduced into mouse zygotes by the CRISPR/Cas system were repaired by the capture of retrotransposons and other genomic DNA, with evidence in some cases of reverse-transcribed mRNA sequences and even exogenous single guide RNA (sgRNA) sequences at DSB sites. RMDR in the mouse zygote was confirmed in at least 2% of the CRISPR-Cas injected mice in this study. Moreover, three alleles were shown to generate novel long-range fusion proteins between *Peg10*-ORF2 and truncated MuERV-L (*Peg10* ORF2-#18) and between *Peg10*-ORF2 and truncated *Pcnt* (*Peg10* ORF2-#2) in the DSB-introduced mice (Supplementary Fig. 8). Therefore, DSB repair by CRISPR-Cas injection into mouse zygotes has the potential to generate novel genes sequences. In nature, DSBs result from both exogenous insults (e.g., reactive oxygen species, irradiation, chemical agents, ultraviolet light) and endogenous cellular events (e.g., transposition, meiotic double strand break formation)^5^,^32^,^33^. Apart from its frequency, extrapolation of our findings here on the consequences of DSBs leads us to conclude that RMDR may contribute to the generation of novel gene sequences under certain natural conditions. In fact, by comparing rodent genomes, we found that two *de novo* MuERV-L insertions in wild-type C57BL/6 mice show features characteristic of RMDR. Although we could not exclude the possibility of DNA recombination, we consider that this finding is compatible with DSB repair by the capture of retrotransposon sequences occurring in natural conditions (Fig. 4e,f). Thus, we propose the hypothesis that RMDR has contributed to the evolution of the mammalian genome.

## Methods

### Animals

All animal studies were conducted in accordance with the guidelines approved by the animal care committee of Tokyo Medical Dental University, Osaka University and National Institute of Health Sciences (NIHS). Animals were allowed access to a standard chow diet and water ad libitum and were housed in a pathogen-free barrier facility with a 12L:12D cycle.

### Plasmid preparation

To construct the pCAG-EGxxFP validation plasmid, the N-terminal and C-terminal EGFP coding regions were PCR-amplified and placed under a ubiquitous CAG promoter with the multicloning sites (MCS) BamHI, NheI, PstI, SalI, EcoRI, and EcoRV. The ~500 bp genomic fragments containing the sgRNA target sequence were PCR-amplified and placed in the MCS of the pCAG-EGxxFP validation plasmid. The plasmids expressing *hCas*9 and sgRNA were prepared by ligating oligos into the BbsI site of pX330 (http://www.addgene.org/42230/). The 20 bp sgRNA recognition sequences are shown below.

*Peg10*-ORF1-sgRNA3 (5′- TGTCTCTACTGTGGCAATGG-3′)

*Peg10*-ORF2-sgRNA4 (5′- GTCCGAGCTATGATTGATTC-3′)

*Cxx1a/b*-sgRNA (5′-ACGGGATGGGGTTCCGCCGA-3′)

*Rgag1*-sgRNA (5′-GTGGTGTGGATGTCACTCCA-3′)

*Ddx3y* -sgRNA (5′-GAAAGATGCCTACAGCAGTTT-3′)

*Rsph6a* -sgRNA (5′-GGCTGGACCTCTGTGGCCAG-3′)

*Spaca5* -sgRNA (5′-GCATGAAGGTCTGCAGCATTG-3′)

The oligo DNAs for co-injection with the plasmid into mouse zygotes are shown below.

#1: AACCTACAGTTACTGCTCCCCAAAACATTCATTCACCCACAAGATTTAGAAACATAAAACGGCATAACTTCGTATAATGTATGCTATACGAAGTTATGCGGGGTGGGGGGGGAAGCTGAGGTCTCCGTGTAAACCTCACAAAGTCCGTAGCTGAAGGCTTC

#2: CTGTCCAAGGAAGAAAAGGAGAGACGCCGCAAAATGAATTTGTGTCTCTACTGGGGCAATGGAGGCCATTTCGCCGACACGTGTCCAGCGAAAGCCTCCAAGAATTCGCCGCCGGGAAACTCCCCGGCCCCGCT

### Production of hCas9 mRNA and Peg10-ORF1-sgRNA

To produce the Cas9 mRNA, the T7 promoter was added to the Cas9 coding region of the pX330 plasmid by PCR amplification as previously reported^39^. The T7-Cas9 PCR product was gel purified and used as the template for *in vitro* transcription (IVT) using the mMESSAGE mMACHINE T7 ULTRA kit (Life Technologies). The T7 promoter was added to the Peg10-ORF1-sgRNA region of the pX330 plasmid by PCR purification using the primers listed below.

Peg10-ORF1-IVT-F (TGTAATACGACTCACTATAGGGTGTCTCTACTGTGGCAATGG), IVT-R (AAAAGCACCGACTCGGTGCC)

The T7-sgRNA PCR product was gel purified and used as the template for IVT using the MEGAshortscript T7 kit (Life Technologies). Both the Cas9 mRNA and Peg10-ORF1-sgRNA were DNAse treated to eliminate template DNA and purified using the MEGAclear kit (Life Technologies), and eluted into RNase-free water.

### PCR and DNA sequencing

Genomic DNAs from the embryonic yolk sac or tail tip of pups were prepared using a Maxwll 16 system (Promega). Identification of the indels induced by DSB repair, were confirmed by PCR and subsequent DNA sequencing. The primers used for these DSB repair confirmations were Peg10-F (5′-GGAAGGTCTCAACCCAGACA-3′)/Peg10-R (5′-GTATCTCACGGTGGTCTCCC-3′), Cxx1a-F (5′-CAAATTACTTTGCTCCACTAACCCT-3′)/Cxx1a-R (5′-ATTCAGGAAGCCGTTGTAATCAT-3′), Cxx1b-F (5′-ATTGGGTAGCACTAAGGATTGTTGA-3′)/Cxx1b-R (5′-AGAGGCCTAGAAGTCCTCATCCT-3′), Rgag1-F (5′-TCTGTCCACACCTCTCATGG-3′)/Rgag1-R (5′-TGTTGCCGCTGTATCAGAAG-3′), Rsph6a-F (5′- AAGAATTCCAGGCAGGGTCCAGGATAGG-3′)/Rsph6a-R (5′- AAGGATCCCCTGGCTGAATATCTCATCC-3′), Ddx3y-F (5′- AAGAATTCCATGCCCTCATCTCAATATCCCATAAGGT-3′)/Ddx3y-R (5′- TTCTGCAGGGATAGCCATTGTTGGACTAGTTGGACA-3′) , Spaca5-F (5′- aaGCTAGCCAGTGTCCTTATCCAATCTTTCTCCCTGC-3′)/Spaca5-R (5′- AAGGATCCCCTGGCTGAATATCTCATCC-3′). Each PCR product was purified with an S-400 spin column (GE Health Care) and sequenced with each F and R primer. The extra PCR bands (RMDR alleles) were extracted with a Gel-purification kit (Qiagen) and sub-cloned into a pGEM-T easy vector (Promega), and sequenced with each F and R primer.

### HEK293T transfection and EGxxFP system

Five hundred ng of the pCAG-EGxxFP-target were mixed with 500 ng of pX330 with/without the sgRNA sequences and then introduced into 4 × 10^5^ HEK293T cells/well in a six well plate using Lipofectamine LTX (Life Technologies). The ratio of EGFP fluorescence positive cells/all cells (Hoechest 33342 positive nucleus) was monitored using a fluorescence microscopy EVOS cell counting system (Life Technologies) 48 hrs after transfection.

### One-cell Embryo Injection

B6D2F1 and C57BL/6J female mice were superovulated and IVF was carried out using B6D2F1 and C57BL/6J male mice sperm, respectively. pX330 plasmids with or without oligo DNA to generate knock in mice or mutant mice were injected into the pronucleus of fertilized eggs at the indicated concentrations. hCas9 mRNA and Peg10-ORF1-sgRNA were injected into the cytoplasm of fertilized eggs at the indicated concentration. The eggs were cultivated in KSOM overnight, then transferred into the oviducts of pseudopregnant ICR females.

### Retrotransposition analysis

Identification of RMDR alleles was performed by the BLASTN program from the NCBI server (http://www.ncbi.nlm.nih.gov/BLAST/) and CENSOR program from the GENETIC INFORMATION RESEARCH INSTITUTE (http://www.girinst.org/censor/index.php)^60^, against the mouse genomes using each of the PCR products from the pX330-injected mice as a query.

### Introduction of DSBs into NIH-3T3 cells with or without the RT-inhibitor

Two μg of the pX330 with/without *Peg10*-ORF1 sgRNA sequences were mixed with 500 ng of pTracer-CMV/Bsd and then introduced into 2 × 10^5^ NIH-3T3 cells/well in a six well plate using Lipofectamine LTX (Life Technologies). 24 hour after transfection, cells separated and cultured under two conditions for 2 days, one containing 10 μg/mL Blasticidin S (Life Technologies) and the other 10 μg/mL Blasticidin S and 50 μg/mL Azidothymidine (AZT) (Sigma). Five days after transfection, cells were collected and genomic DNA was extracted. Subsequent PCR products with 1500 and 15 bp internal markers were resolved and quantified by using the Agilent DNA 1000 kit (Agilent Technologies).

### Identification of natural RMDR alleles

Identification of truncated MuERV-L sequences was performed using the BLASTN program (http://www.ncbi.nlm.nih.gov) against the mouse genome using full length MuERV-L (GenBank ID. Y12713) as a query. Among the truncated MuERV-L sequences, two MuERV-L sequences were identified as a murine specific insertion by comparing the sequences with other rodent genomes.

## Additional Information

**How to cite this article**: Ono, R. *et al.* Double strand break repair by capture of retrotransposon sequences and reverse-transcribed spliced mRNA sequences in mouse zygotes. *Sci. Rep.* **5**, 12281; doi: 10.1038/srep12281 (2015).

## Supplementary Material

Supplementary Information
