# Supplementary material for: Double strand break repair by capture of retrotransposon sequences and reverse-transcribed spliced mRNA sequences in mouse zygotes
Source: Sci Rep. 2015 Jul 28;5:12281. doi: 10.1038/srep12281 (PMC4516963; doi:10.1038/srep12281)
Supplement: Supplementary Information [file srep12281-s1.pdf]

Double strand break repair by capture of retrotransposon sequences and reverse-transcribed spliced mRNA sequences in mouse zygotes

Authors: Ryuichi Ono[1, 2]*†, Masayuki Ishii[2]†, Yoshitaka Fujihara[3], Moe Kitazawa[2], Takako Usami[4], Tomoko Kaneko-Ishino[5], Jun Kanno[1], Masahito Ikawa[3], and Fumitoshi Ishino[2, 6]

Affiliations:

[1] Division of Cellular & Molecular Toxicology, Biological Safety Research Center, National Institute of Health Sciences (NIHS), 1-18-1 Kamiyoga, Setagaya-ku, Tokyo, 158-8501, Japan

[2] Department of Epigenetics, Medical Research Institute, Tokyo Medical and Dental University, 1-5-45 Yushima, Bunkyo-ku, Tokyo 113-8510, Japan.

[3] Research Institute for Microbial Diseases, Osaka University, Suita, Osaka 565-0871, Japan.

[4] Facility for Recombinant Mice, Medical Research Institute, Tokyo Medical and Dental University, 2-3-10 Kandasurugadai, Chiyoda-ku, Tokyo 101-0062, Japan.

[5] School of Health Sciences, Tokai University, Bohseidai, Isehara, Kanagawa 259-1193, Japan.

[6] Global Center of Excellence Program for International Research Center for Molecular Science in Tooth and Bone Diseases, Tokyo Medical and Dental University, 1-5-45 Yushima, Bunkyo-ku, Tokyo 113-8510, Japan.

*Correspondence to: R.O. (onoryu@nihs.go.jp).

†These authors contribute equally.

Supplementary Figures 1-8

Supplementary Tables 1-3

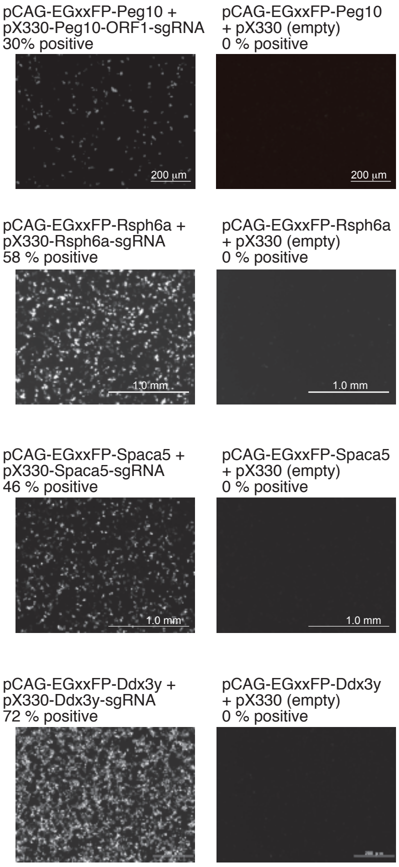

Supplementary Figure 1. Validation of efficiency of sgRNAs. The efficiency of DSB-mediated homology dependent repair was validated by observing EGFP fluorescence 48 hrs after the transfection (pX330 without sgRNA (negative control) and pX330 with *Peg10*-ORF1-sgRNA, *Rsph6a*-sgRNA, *Spaca5*-sgRNA, and *Ddx3y*-sgRNA with each pCAG-EGxxFP plasmid. (Scales are indicated in each panel). The percentages of EGFP-positive cells are indicated.

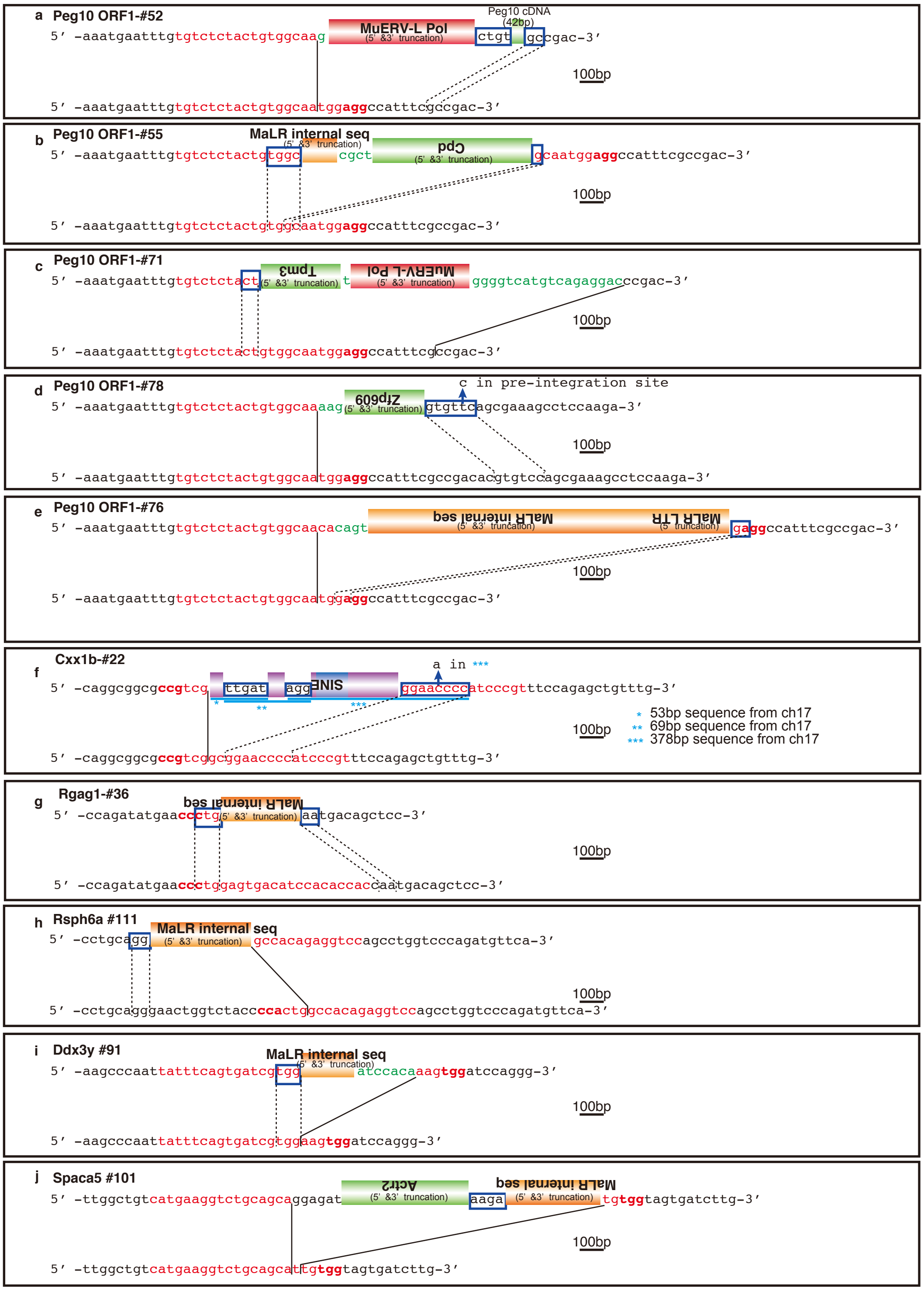

Supplementary Figure 2. Structure of the captured DNA sequences induced by DSBs. *De novo* inserted retrotransposons and mRNAs at the *Peg10*-ORF1 (**a**-**e**), *Cxx1b* (**f**), *Rgag1* (**g**), *Rshph6a* (**h**), *Ddx3y* (**i**) and *Spaca5* (**j**) loci were induced by pX330 plasmids with or without oligo DNA, or by sgRNA and *hCas9* mRNA injection into mouse zygotes. Both the post-integration site and pre-integration sequences (bottom of the panel) are shown. The nucleotide sequences corresponding to the single guide RNA sequence and the PAM sequences are shown in red and bold red characters, respectively. Black lines indicate the junction sites between pre- and post-integration sequences. The sequences in the blue boxes are overlapping microhomologies and are marked with black dotted lines. Short sequences of unknown origin are shown in green. (**a**) Truncated MuERV-L together with a partial *Peg10* exon sequence were inserted as single 4bp and 2bp microhomologies. (**b**) Truncated MaLR together with the *Cpd* exon sequence were inserted as 4bp and 1bp microhomologies. (**c**) Truncated MuERV-L together with a partial *Tpm3* exon sequence were inserted as 2bp microhomologies. (**d**) A partial *Zfp609* exon sequence was inserted as a 6bp microhomology (1bp mismatch). (**e**) A truncated MaLR internal sequence was inserted as a 2bp microhomology. (**f**) Three genomic fragments, including SINEs, were inserted as 8bp (1bp mismatch), 5bp and 3bp microhomologies. (**g**) A truncated MaLR internal sequence was inserted as 3bp and 2bp microhomologies. (**h**) A truncated MaLR internal sequence was inserted as a 2bp microhomology. (**i**) A truncated MaLR internal sequence was inserted as a 3bp microhomology. (**j**) A truncated MaLR internal sequence together with a partial *Actr2* exon sequence were inserted as a single 4bp overlapping microhomology.

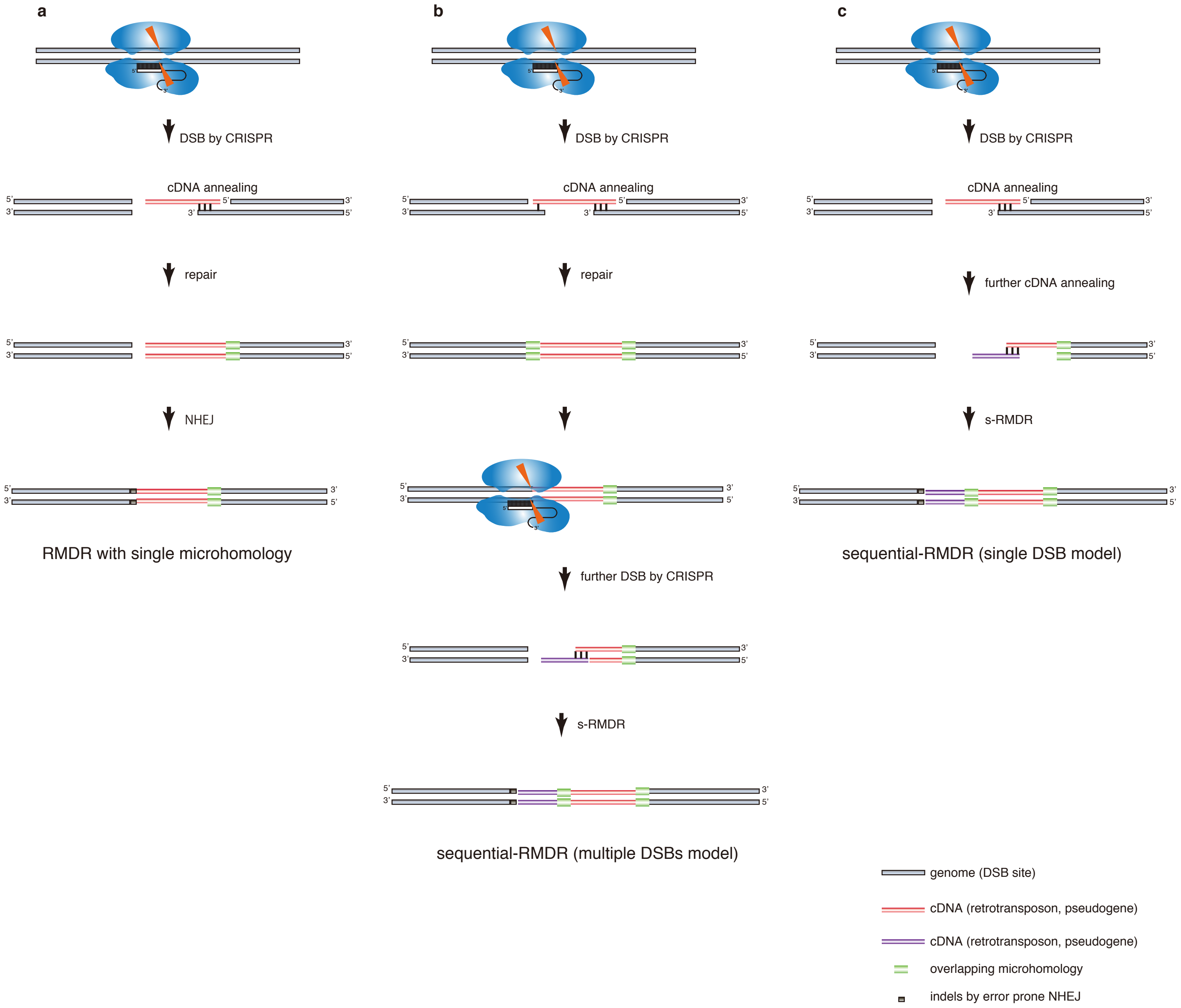

Supplementary Figure 3. Possible mechanisms of sequential-RMDR. DSBs (orange triangles) induced by CRISPR-CAS (blue sphere) are shown being repaired by RMDR with a single microhomology (**a**) and s-RMDR (sequential-RMDR) (**b, c**). (**a**) A cDNA (red bar) generated by RT anneals with one of the two DSB DNA ends, while the other end is repaired by NHEJ (RMDR with a single microhomology). (**b**) A cDNA (red bar) generated by RT anneals with one of two DSB DNA ends, while the other end is repaired with intact target recognition sequence. Further DSB is introduced and another cDNA (purple bar) is captured with s-RMDR (multiple DSBs model). (**c**) A cDNA (red bar) anneals with one of the two DSB DNA ends and the other end anneals with the other cDNA (purple bar) and is repaired by s-RMDR (single DSB model).

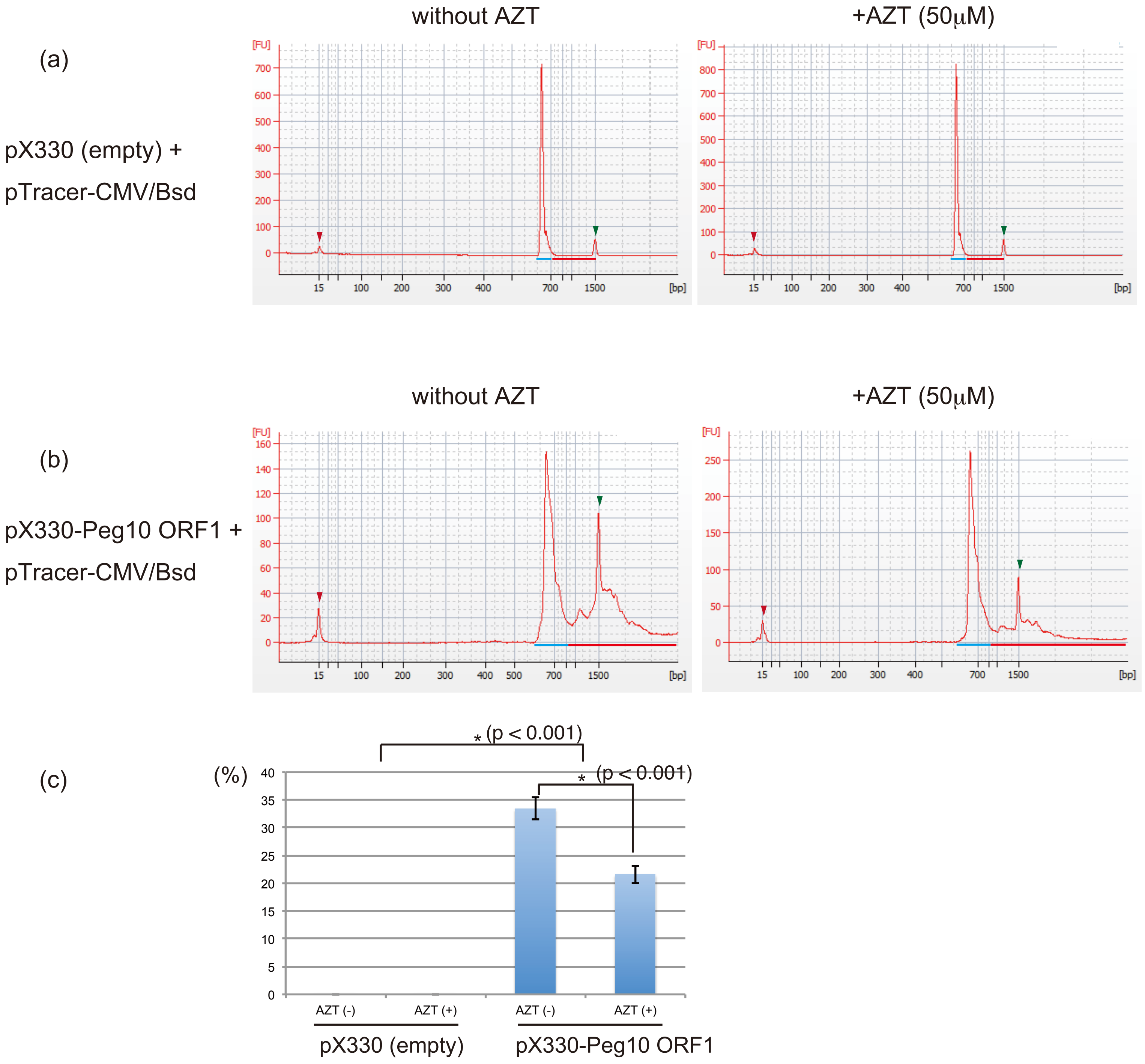

Supplementary Figure 4. DSB repair by the capture of long DNA sequences was induced by CRISPR/Cas plasmid transfection in NIH-3T3 cells and was partially inhibited by the RT-inhibitor AZT. Electrophoresis of the PCR products from each of the pX330 plasmid-transfected NIH-3T3 cell lines was performed using a Bioanalyzer DNA 1000 tip. The PCR products from each of the pX330 plasmid-transfected NIH-3T3 cell lines with 1500 (green arrow) and 15 bp (red arrow) internal markers were resolved and quantified. (**a-c**) (**a**) There was a single sharp PCR product detected at approximately 655 bp (expected length, blue bar) in pX330 (empty) transfected cells with or without AZT. No peaks were detected over 700 bp (red bar). (**b**) Broadened PCR peaks approximately 655 bp (expected length, blue bar) and larger PCR products (red bar) were detected in pX330-*Peg10*-ORF1 plasmid transfected cells with or without AZT. (**c**) The ratio (molarity) of the PCR products with the captured DNA sequences (red bar) was quantified (red bar/red bar + blue bar) (N=3). Capture of the long DNA sequences was inhibited by the addition of the RT-inhibitor AZT.

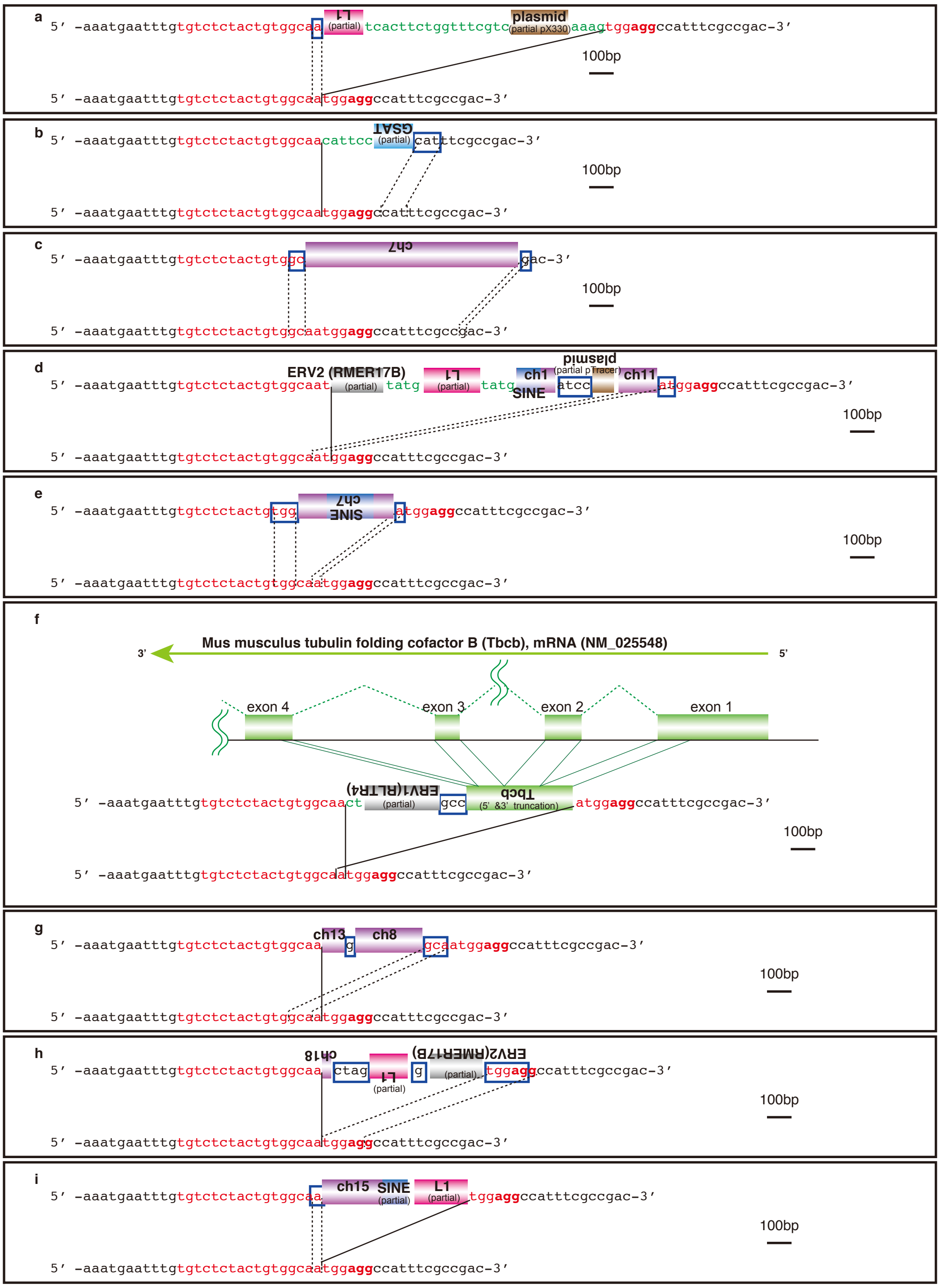

Supplementary Figure 5. To be continued

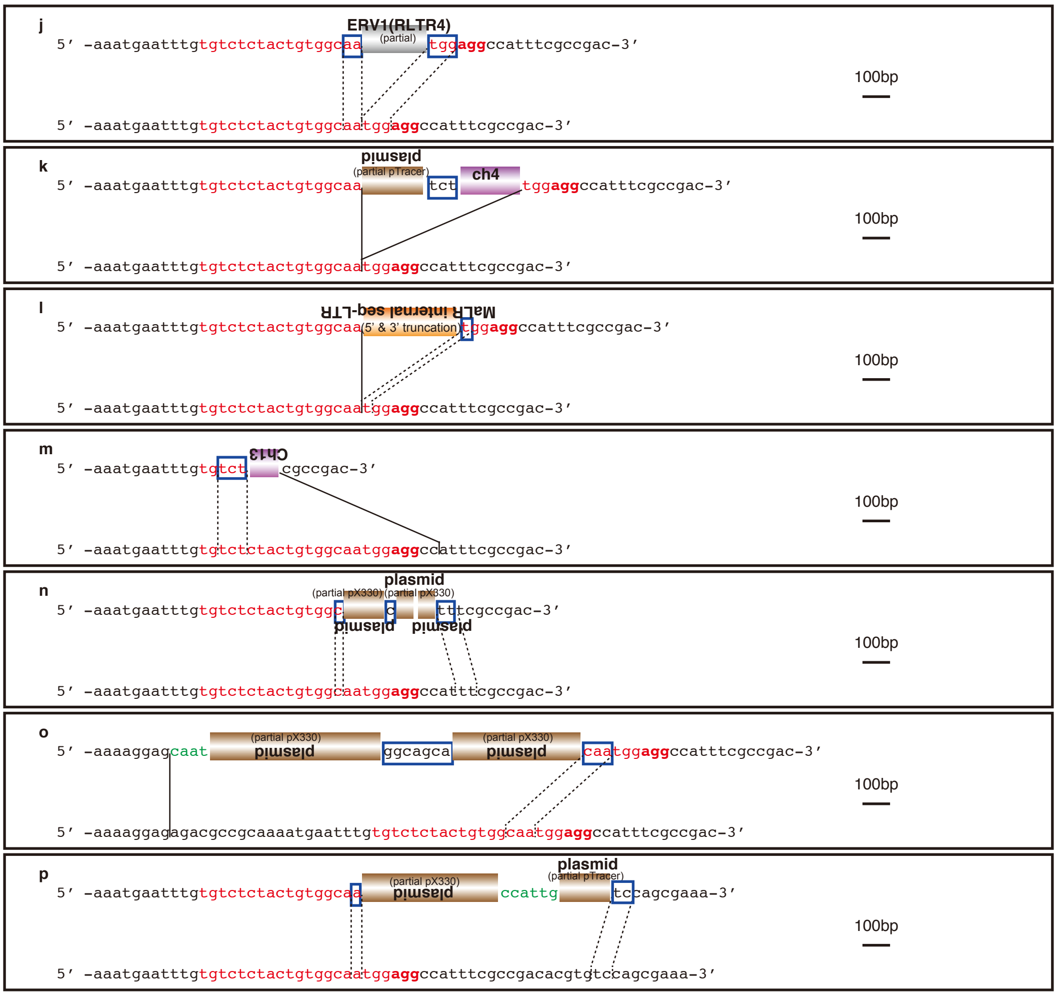

Supplementary Figure 5. Structure of the captured DNA sequences induced by DSBs in the NIH-3T3 cell line without AZT. *De novo* inserted retrotransposons, genomic DNA, mRNA and plasmid DNA sequences at the *Peg10*-ORF1 locus were induced by CRISPR/Cas plasmid transfection in NIH-3T3 cells. Both the post-integration site and pre-integration sequences (bottom of the panel) are shown. The nucleotide sequences corresponding to the single guide RNA sequence and the PAM sequences are shown in red and bold red characters, respectively. Black lines indicate the junction sites between pre- and post-integration sequences. The sequences in the blue boxes are overlapping microhomologies and are marked with black dotted lines. Short sequences of unknown origin are shown in green. (**a**) A truncated L1 together with a partial plasmid DNA sequence was inserted with microhomologies. (**b**) A partial mouse gamma-satellite repetitive sequence (GSAT) was captured with a single microhomology. (**c**) Genomic sequence from chromosome 7 was captured. (**d**) Partial ERV2, L1, genomic DNA from chromosome1 with SINE, plasmid DNA and genomic DNA from chromosome 11 were captured with microhomologies. (**e**) Genomic DNA from chromosome 7, which has a full length SINEB2, was captured. (**f**) A truncated ERV1 retrotransposon and processed mRNA sequence of tubulin folding cofactor B (Tbcb) were captured. (**g**) Two genomic DNA sequences from chromosome 13 and 8 were captured with two microhomologies. (**h**) A genomic DNA sequence from chromosome 18, truncated L1, and ERV2 were captured with three microhomologies. (**i**) Genomic DNA from chromosome 15, which has a truncated SINEB2 sequence and a truncated L1, were captured. (**j**) A truncated ERV1 was captured with two microhomologies. (**k**) Plasmid DNA sequence and genomic DNA from chromosome 4 were captured. (**l**) A truncated MaLR and genomic DNA from chromosome 4 were captured. (**m**) Genomic DNA from chromosome 13 was captured. (**n-P**) Double to triple plasmid DNA sequences were captured.

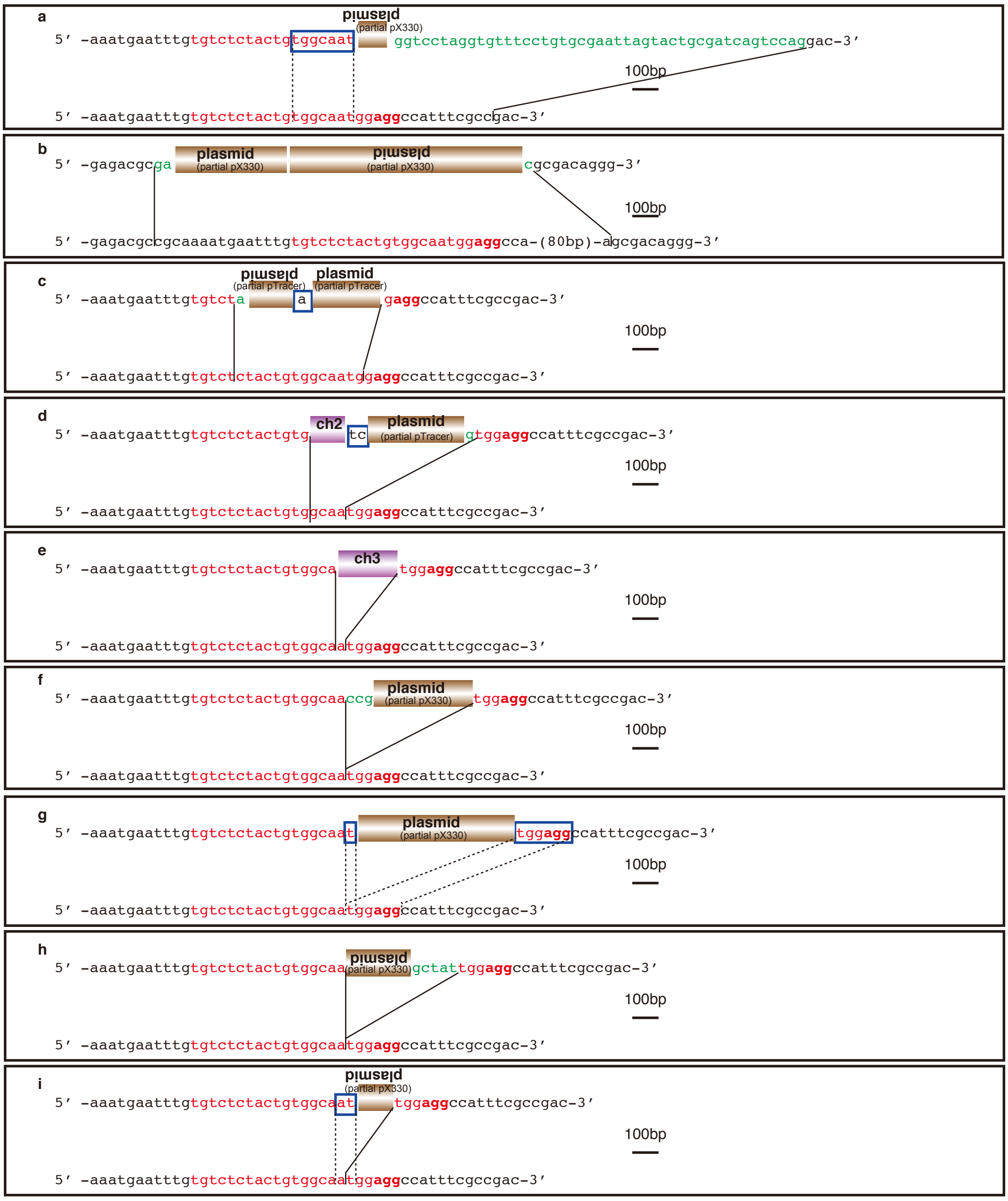

Supplementary Figure 6. To be continued

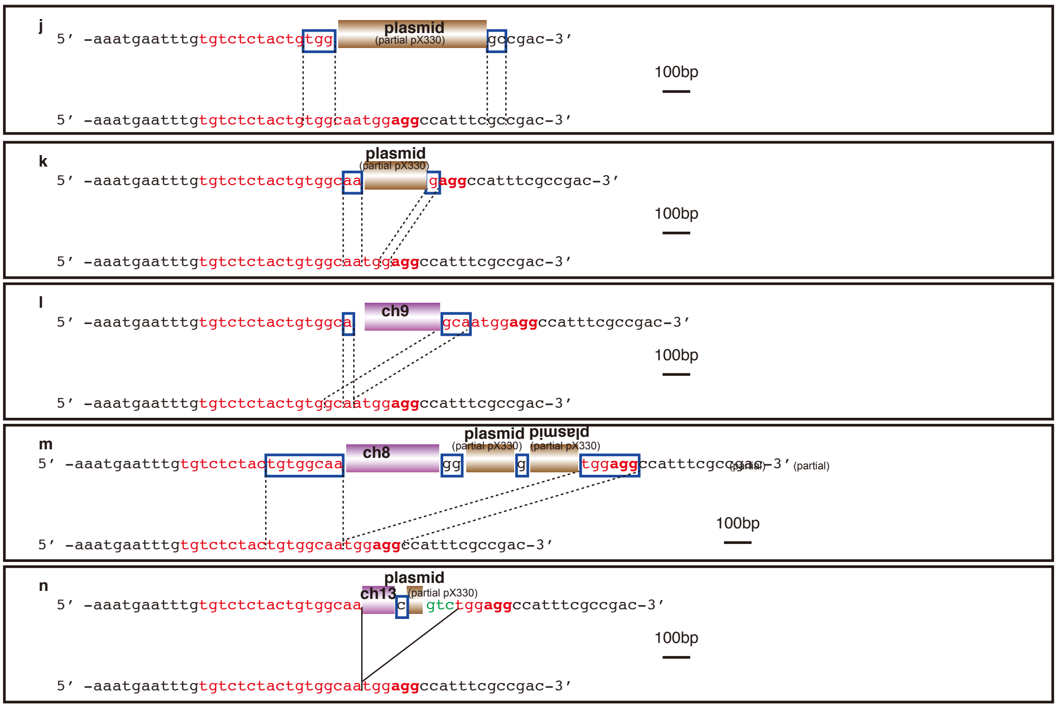

Supplementary Figure 6. Structure of the captured DNA sequences induced by DSBs in the NIH-3T3 cell line with AZT. *De novo* inserted genomic DNA and plasmid DNA sequences at the *Peg10*-ORF1 locus were induced by CRISPR/Cas plasmid transfection in NIH-3T3 cells with the RT-inhibitor AZT. Both the post-integration site and pre-integration sequences (bottom of the panel) are shown. The nucleotide sequences corresponding to the single guide RNA sequence and the PAM sequences are shown in the red and bold red characters, respectively. Black lines indicate the junction sites between pre- and post-integration sequences. The sequences in the blue boxes are overlapping microhomologies and are marked with black dotted lines. Short sequences of unknown origin are shown in green. Single plasmid DNA was captured with or without microhomologies (**a**, **f**, **g**, **h**, **i**, **j**, and **k**) (**b, c**) Double plasmid DNAs were captured. (**d**) Genomic DNA from chromosome 2 and plasmid DNA were captured. (**e**) Genomic DNA sequence from chromosome 3 was captured. (**l**) Genomic DNA from chromosome 9 was captured. (**m**) Genomic DNA from chromosome 8 and two plasmid DNAs were captured with 4 microhomologies. (**n**) Genomic DNA from chromosome 13 and plasmid DNA were captured.

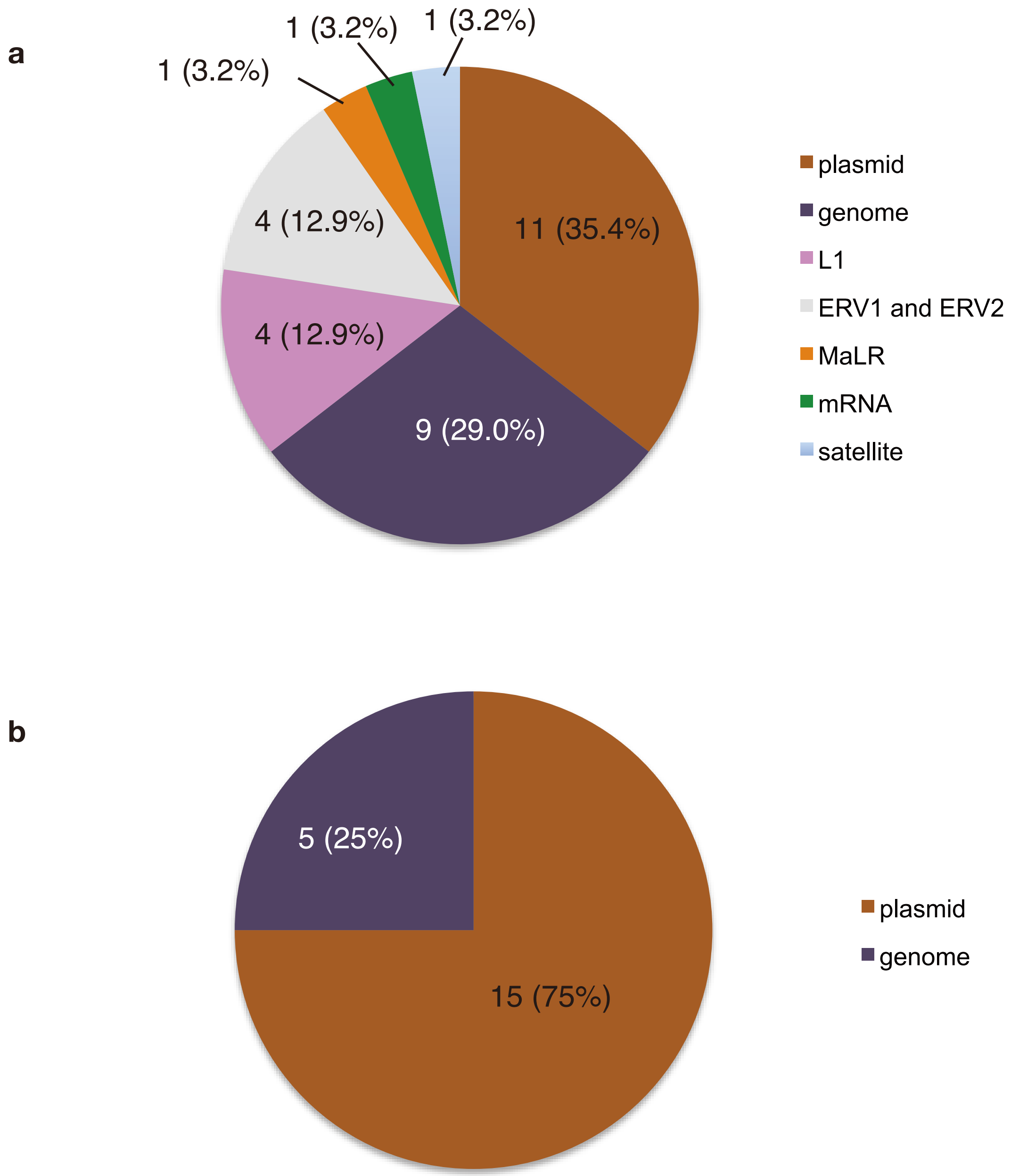

Supplementary Figure 7. Distribution of captured DNA sequences at DSB sites in the NIH-3T3 cell line with or without the RT-inhibitor AZT. (**a**) Distribution of 31 insertion sequences at CRISPR/Cas DSB sites from 16 alleles of NIH-3T3 cells without AZT. (**b**) Distribution of 20 insertion sequences at CRISPR/Cas DSB sites from 14 alleles of NIH-3T3 cells with AZT (50μM).

Peg10 WT

a

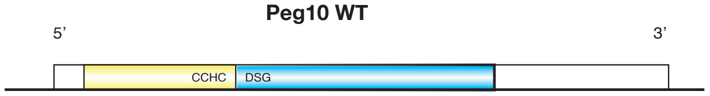


```
        10         20         30         40         50         60         70         80         90        100
MAAAGGSSNC PPPPPPPPPN NNNNNNTPKS PGVPDAEDDD ERRHDELPED INNFDEDMNR QFENMNLLDQ VELLAQSYSL LDHLDDFDDD DEDDDFDPEP
       110        120        130        140        150        160        170        180        190        200
DQDELPEYSD DDDLELQGAA AAPIPNFFSD DDCLEDLPEK FDGNPDMLGP FMYQCQLFME KSTRDFSVDR IRVCFVTSML IGRAARWATA KLQRCTYLMH
       210        220        230        240        250        260        270        280        290        300
NYTAFMMELK HVFEDPQRRE AAKRKIRRLR QGPGPVVDYS NAFQMIAQDL DWTEPALMDQ FQEGLNPDIR AELSRQEAPK TLAALITACI HIERRLARDA
       310        320        330        340        350        360        370        380        390        400
AAKPDPSPRA LVMPPNSQTD PTEPVGGARM RLSKEEKERR RKMNLCLYCG NGGHFADTCP AKASKNSPPG KLPGPAVGGP SATGPERIRS PPSEASTQHL
       410        420        430        440        450        460        470        480        490        500
QVMLQIHMPG RPTLFVRAMI DSGASGNFID QDFVIQNAIP LRIKDWPVMV EAIDGHPIAS GPIILETHHL IVDLGDHREI LSFDVTQSPF FPIVLGIRWL
       510        520        530        540        550        560        570        580        590        600
STHDPHITWS TRSIVFNSDY CRLRCRMFAQ IPSNLLFTVP QPNLHPYLLH HVHPHVHPHM HQHLHQHLHQ FLHPDPHQYP HPDPHYHHHQ QADMQHQLQQ
       610        620        630        640        650        660        670        680        690        700
YLYQYLYYHL YPVMHHHLPP DQHEHLHEYL HQYLHQYLHQ FLHHHLHPDL HQYLYQYLHN HMNPDPHHHP HPDLPQDPHH PPHQDPHQHP DPHQDPPHQD
       710        720        730        740        750        760        770        780        790        800
PHQDAHQDPH MDPHLHQHQH PQPQPHPQQH PNHPQQPPFF YHMAGFRIYH PVRYYYIQNV YTPVDEHVYP GHRVVDPNIE MIPGAHSLPS GHLYSMSESE
       810        820        830        840        850        860        870        880        890        900
MNALRNFVDR NVKDGLMTPT VAPNGAQVLQ VKRGWKLQVT YNCRAPQSGT IQNQYLRMSL PNMGDPAHLA SYGEFVQVPG YPYPAYVYYT SPHMMTAWYP
       910        920        930        940        950        960        970        980        990       1000
VGRDVHGRII VVPVVITWSQ NTNRQPPVPQ YPPPQPPPPP PPPPPPPPPP PASSCSAA
```

Peg10 #18

b

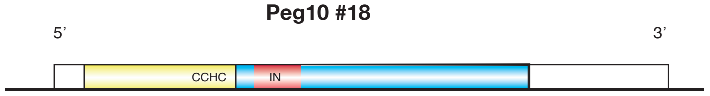


```
        10         20         30         40         50         60         70         80         90        100
MAAAGGSSNC PPPPPPPPPN NNNNNNTPKS PGVPDAEDDD ERRHDELPED INNFDEDMNR QFENMNLLDQ VELLAQSYSL LDHLDDFDDD DEDDDFDPEP
       110        120        130        140        150        160        170        180        190        200
DQDELPEYSD DDDLELQGAA AAPIPNFFSD DDCLEDLPEK FDGNPDMLGP FMYQCQLFME KSTRDFSVDR IRVCFVTSML IGRAARWATA KLQRCTYLMH
       210        220        230        240        250        260        270        280        290        300
NYTAFMMELK HVFEDPQRRE AAKRKIRRLR QGPGPVVDYS NAFQMIAQDL DWTEPALMDQ FQEGLNPDIR AELSRQEAPK TLAALITACI HIERRLARDA
       310        320        330        340        350        360        370        380        390        400
AAKPDPSPRA LVMPPNSQTD PTEPVGGARM RLSKEEKERR RKMNLCLYCG NGGHFADTCP AKASKNSPPG KLPGPAVGGP SATGPERIRS PPSEASTQHL
       410        420        430        440        450        460        470        480        490        500
QVMLQIHMPG RPTLFVRAMI VLTGVDTYSG YGFAFPARNA SAKTTIHGLT ECLIYRHGIP HSIASDQGTH FTAREVRQWA HDHGIHWSYH IPHHAEAAGL
       510        520        530        540        550        560        570        580        590        600
IERWNGLLKT QLQRQLGGNS LEGWGRVLQK AVYALNQRCS GASGNFIDQD FVIQNAIPLR IKDWPVMVEA IDGHPIASGP IILETHHLIV DLGDHREILS
       610        620        630        640        650        660        670        680        690        700
FDVTQSPFFP IVLGIRWLST HDPHITWSTR SIVFNSDYCR LRCRMFAQIP SNLLFTVPQP NLHPYLLHHV HPHVHPHMHQ HLHQHLHQFL HPDPHQYPHP
       710        720        730        740        750        760        770        780        790        800
DPHYHHHQQA DMQHQLQQYL YQYLYYHLYP VMHHHLPPDQ HEHLHEYLHQ YLHQYLHQFL HHHLHPDLHQ YLYQYLHNHM NPDPHHHPHP DLPQDPHHPP
       810        820        830        840        850        860        870        880        890        900
HQDPHQHPDP HQDPPHQDPH QDAHQDPHMD PHLHQHQHPQ PQPHPQQHPN HPQQPPFFYH MAGFRIYHPV RYYYIQNVYT PVDEHVYPGH RVVDPNIEMI
       910        920        930        940        950        960        970        980        990       1000
PGAHSLPSGH LYSMSESEMN ALRNFVDRNV KDGLMTPTVA PNGAQVLQVK RGWKLQVTYN CRAPQSGTIQ NQYLRMSLPN MGDPAHLASY GEFVQVPGYP
      1010       1020       1030       1040       1050       1060       1070       1080       1090       1100
YPAYVYYTSP HMMTAWYPVG RDVHGRIIVV PVVITWSQNT NRQPPVPQYP PPQPPPPPPP PPPPPPPPPA SSCSAA
```

Peg10 #2

c

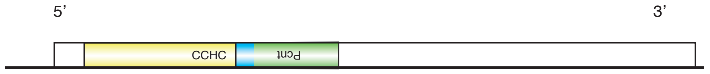


```
        10         20         30         40         50         60         70         80         90        100
MAAAGGSSNC PPPPPPPPPN NNNNNNTPKS PGVPDAEDDD ERRHDELPED INNFDEDMNR QFENMNLLDQ VELLAQSYSL LDHLDDFDDD DEDDDFDPEP
       110        120        130        140        150        160        170        180        190        200
DQDELPEYSD DDDLELQGAA AAPIPNFFSD DDCLEDLPEK FDGNPDMLGP FMYQCQLFME KSTRDFSVDR IRVCFVTSML IGRAARWATA KLQRCTYLMH
       210        220        230        240        250        260        270        280        290        300
NYTAFMMELK HVFEDPQRRE AAKRKIRRLR QGPGPVVDYS NAFQMIAQDL DWTEPALMDQ FQEGLNPDIR AELSRQEAPK TLAALITACI HIERRLARDA
       310        320        330        340        350        360        370        380        390        400
AAKPDPSPRA LVMPPNSQTD PTEPVGGARM RLSKEEKERR RKMNLCLYCG NGGHFADTCP AKASKNSPPG KLPGPAVGGP SATGPERIRS PPSEASTQHL
       410        420        430        440        450        460        470        480        490        500
QVMLQIHMPG RPTLFVRAMI AAFAAERAAL VSLGGHRPPA SCGSAALSGG ARAVGPLRAR VSGSAQPPGS SAALRAGPSP VPATLRSARP RSAAPSAGCL
       510        520        530        540        550        560        570        580        590        600
WPHARVLKPH TVGCFEQAAS PKIVSTPPGP WSSGPPSAGT AVAAPPAGPG GPAPFRAQTP APPAQPSAPS PSRPAQL
```

Supplementary Figure 8. Schematic representation of the Peg10-MuERV-L fusion protein and Peg10-PcntAS fusion protein foemed by the capture of retrotransposons at DSB sites. (**a**) Peg10 ORF1 and ORF2 protein sequences are shown in yellow and blue bars, respectively. (**b**) The 852-973 a.a. region of MuERV-L Pol proteins (CAA73251, red bar), containing an integrase domain, was inserted into the Peg10 ORF2 protein, donating a novel integrase domain to the Peg10 ORF1-2 fusion protein sequence. (**c**) The *Pcnt* gene 7599-6802bp (NM_001282992, green bar) was inserted into the Peg10 ORF2 protein in an antisense orientation, generating a novel protein coding pattern.

plasmid name/concentration	zygote	injection #	transfer #	born or dissected	wt	mutant (mosaicism)	mutant (null)	knock-in(KI) (mosaicism)	mutatant rate	Capture of long DNA sequences	ratio of mice with the capture of long DNA sequences	stage	mice number
Peg10-sgRNA4-pX330/1ng + oligo1	BDF1	120	84	4	3	1	0	0	25.0%	1	25.0%	newborn pups	#1-#4
Peg10-sgRNA4-pX330/1.6ng	BDF1	132	67	1	0	1	0	-	100.0%	0	0.0%	newborn pups	#5
Peg10-sgRNA4-pX330/1.6ng	BDF1	132	20	4	0	0	4	-	100.0%	1	25.0%	10.5dpc embyos	#6-#9
Peg10-sgRNA4-pX330/1.6ng	C57Bl/6	129	83	9	5	4	0	-	44.4%	2	22.2%	newborn pups	#10-#18
Peg10-sgRNA4-pX330 total		513	254	18	8	6	4	0	55.6%	4	22.2%		

plasmid name/concentration	zygote	injection #	transfer #	born	wt	mutant (mosaicism)	mutant (null)		mutation rate	Capture of long DNA sequence	ratio of mice with the capture of long DNA sequences	stage	mice number
Cxx1a/b-sgRNA5-pX330 2.5ng	C57Bl/6	343	252	7	4	1	2		42.9%	1	14.3%	newborn pups	#21-#27

plasmid name/concentration	zygote	injection #	transfer #	born	wt	mutant (mosaicism)	mutant (null)		mutation rate	Capture of long DNA sequence	ratio of mice with the capture of long DNA sequences	stage	mice number
Cxx1a/b-sgRNA5-pX330 2.5ng	C57Bl/6	343	252	7	4	0	3		42.9%	2	28.6%	newborn pups	#21-#27

plasmid name/concentration	zygote	injection #	transfer #	born	wt	mutant (mosaicism)	mutant (null)		mutation rate	Capture of long DNA sequence	ratio of mice with the capture of long DNA sequences	stage	mice number
Rgag1-sgRNA1-pX330 2.5ng	C57Bl/6	390	309	14	6	3	5		57.1%	2	14.3%	newborn pups	#31-#44

plasmid name/concentration	zygote	injection #	transfer #	born	wt	mutant (mosaicism)	mutant (null)	knock-in(KI) (mosaicism)	mutation rate	Capture of long DNA sequence	ratio of mice with the capture of long DNA sequences	stage	mice number
Peg10-sgRNA2-pX330/1ng + oligo2	BDF1	152	94	12	0	12	0	5	100.0%	5(only 3 were successfully sequenced)	41.7%	newborn pups	#51-#66

plasmid name/concentration	zygote	injection #	transfer #	born	wt	mutant (mosaicism)	mutant (null)		mutation rate	Capture of long DNA sequence	ratio of mice with the capture of long DNA sequences	stage	mice number
Ddx3y-sgRNA(3+4)-pX330 / 5ng	BDF1	125	47	5 (♀3, ♂2)	0	-	2		100.0%	1	50.0%	newborn pups	#91-#95

plasmid name/concentration	zygote	injection #	transfer #	born	wt	mutant (mosaicism)	mutant (null)		mutation rate	Capture of long DNA sequence	ratio of mice with the capture of long DNA sequences	stage	mice number
Spaca5-sgRNA2-pX330 / 5ng	BDF1	70	57	7	4	2	1		42.9%	1	14.3%	newborn pups	#101-107

plasmid name/concentration	zygote	injection #	transfer #	born	wt	mutant (mosaicism)	mutant (null)		mutation rate	Capture of long DNA sequence	ratio of mice with the capture of long DNA sequences	stage	mice number
Rsph6a-sgRNA4-pX330 / 5ng	BDF1	158	99	13	10	1	2		23.1%	1	7.7%	newborn pups	#111-#123

Supplementary Table 1 Generation of mutant and knock-in mice via pX330 plasmid injection. pX330 plasmids containing Peg10-ORF1-sgRNA, Peg10-ORF2-sgRNA, Cxx1a/b-sgRNA, Rgag1-sgRNA, Ddx3y-sgRNA, Spaca5-sgRNA and Rsph6a-sgRNA were injected into BDF1 or C57BL/6 mouse zygotes at the indicated concentrations. For the production of knock-in mice, 10 ng/μl oligo DNA was added to the pX330 plasmid solutions. The mutations were identified by sequencing the PCR products.

sgRNA, Cas9mRNA name/concentration	zygote	injection #	transfer #	born	wt	mutant (mosaicism)	mutant (null)	mutation rate	Capture of long DNA sequence	ratio of mice with the capture of long DNA sequences	stage	mice number
Peg10-sgRNA2/25ng + Cas9mRNA/50ng	BDF1	151	98	13	0	13	0	100.0%	5	38.5%	newborn pups	#71-#83

Supplementary Table 2 Generation of mutant mice via sgRNA and Cas9 mRNA injection. Peg10-ORF1-sgRNA and Cas9 mRNA were injected into BDF1 mouse zygotes at the indicated concentration. The injected eggs were transferred into pseudopregnant females. The mutations were identified by sequencing PCR products.

MuERV-L star	MuERV-L end		Chromosome	Start	End	Ori	E-Val	%ID	Length	TSD
1	6471	+	Chr:16	57805225	57811694	+	0.00E+00	98.24	6471	CCGTC
1	6471	+	Chr:6	77700452	77706922	+	0.00E+00	98.24	6472	AAGAA
1	6471	+	Chr:2	113095789	113102258	+	0.00E+00	98.18	6471	GGTCT
1	6471	+	Chr:17	27281585	27288053	+	0.00E+00	98.19	6471	ACGCC
1	6471	−	Chr:9	93394812	93401281	+	0.00E+00	98.13	6471	GATAC
1	6471	−	Chr:3	62356899	62363368	+	0.00E+00	98.11	6471	GGGTG
1	6471	+	Chr:8	107732118	107738587	+	0.00E+00	98.08	6471	GGTGG
1	6471	−	Chr:3	79046229	79052711	+	0.00E+00	97.98	6483	ATTAT
1	6471	+	Chr:5	94847059	94853528	+	0.00E+00	98.02	6471	ATAGG
1	6471	+	Chr:17	33867142	33873611	+	0.00E+00	98.04	6471	CCAGG
1	6471	−	Chr:2	114180872	114187339	+	0.00E+00	98.08	6471	GAGGA/CAGGA (1bp mismatch)
1	6471	+	Chr:9	114219046	114225515	+	0.00E+00	98.04	6471	GTACC
1	6471	+	Chr:5	109064593	109071061	+	0.00E+00	98.05	6471	GTATG
1	6471	+	Chr:1	152477534	152484003	+	0.00E+00	97.96	6471	CAGGGC 6bp
1	6471	−	Chr:9	96104893	96111360	+	0.00E+00	98.07	6471	CTCTG
1	6471	−	Chr:1	82396584	82403045	+	0.00E+00	98.05	6471	CACAG
1	6471	−	Chr:1	188923410	188929877	+	0.00E+00	97.99	6471	GAGGC
1	6471	−	Chr:13	62726497	62732966	+	0.00E+00	97.88	6471	ATAGT
1	6471	+	Chr:2	99050973	99057454	+	0.00E+00	97.86	6483	CCAGT
1	6471	−	Chr:9	62861832	62868299	+	0.00E+00	97.91	6471	GTGCT
1	6471	+	Chr:7	65260088	65266557	+	0.00E+00	97.87	6471	GTATG
1	6471	+	Chr:6	44106670	44113139	+	0.00E+00	97.88	6471	GCTAC
1	6471	−	Chr:1	13207528	13214007	+	0.00E+00	97.83	6483	CAGAG
1	6471	−	Chr:11	105847886	105854355	+	0.00E+00	97.82	6471	GGGAC
1	6471	−	Chr:2	140345880	140352349	+	0.00E+00	97.84	6471	ACATC
1	6471	+	Chr:9	112330046	112336513	+	0.00E+00	97.96	6471	GTATG
1	6471	−	Chr:19	61168774	61175244	+	0.00E+00	97.88	6472	CACAG/CACAT (1bp mismatch)
1	6471	+	Chr:1	98766178	98772659	+	0.00E+00	97.76	6483	CACAC
1	6471	+	Chr:18	3053253	3059720	+	0.00E+00	97.87	6471	AGTAA
1	6471	+	Chr:5	78140969	78147437	+	0.00E+00	97.77	6471	CTCTC
1	6471	+	Chr:17	53747682	53754149	+	0.00E+00	97.68	6471	CCAAG
1	6471	−	Chr:4	80562981	80569450	+	0.00E+00	97.67	6471	ATTGG
1	6471	−	Chr:2	132885603	132892072	+	0.00E+00	97.64	6471	GCAAG
1	6471	−	Chr:10	19729545	19736015	+	0.00E+00	97.54	6472	CATGG
1	6471	−	Chr:10	22533274	22539758	+	0.00E+00	97.5	6486	GGAGA
1	6471	−	Chr:7	21244735	21251204	+	0.00E+00	97.4	6471	GCTGG/GCCGG (1bp mismatch)

Supplementary Table 3 Analysis of MuERV-L TSD sequences. Endogenous full length MuERV-L sequences were identified by the BLAST program in the mouse genome, and their TSD sequences are shown. The MuERV-Ls exhibit random 5bp TSD sequences.
